# Dense retrieval and reranking for referenced provisions in electric power audit systems

**DOI:** 10.1371/journal.pone.0344683

**Published:** 2026-03-13

**Authors:** Qinglin Meng, Ying He, Sheharyar Hussain, Fei Zhou, Jianbin Xu, Guanqiao Zhao, Deyi Xiong

**Affiliations:** 1 Green Power Research Institute, Tianjin Renai College, Tianjin, China; 2 College of Intelligence and Computing, Tianjin University, Tianjin, China; 3 School of Electrical and Information Engineering, Tianjin University, Tianjin, China; 4 State Grid Tianjin Electric Power Company, State Grid Corporation of China, Tianjin, China; 5 China Electric Power Research Institute, State Grid Corporation of China, Beijing, China; 6 Tianjin Electric Power Technology Development Co., Ltd, State Grid Corporation of China, Tianjin, China; Beijing University of Technology, CHINA

## Abstract

Electric power audits require practitioners to describe an audit issue and justify the final opinion by citing an appropriate referenced provision. In practice, the referenced provision should be retrieved from an authoritative provision corpus rather than generated, because correctness and traceability are critical in audit workflows. This paper proposes a dense retrieval and reranking framework for referenced provision retrieval in electric power audit systems. The method follows a two-stage pipeline: a two-tower dense retriever efficiently recalls a small candidate set (top-20) from a large provision corpus, and a one-tower scoring model performs fine-grained reranking by jointly modeling the audit problem description and each candidate provision. To strengthen semantic matching under audit-specific contexts, the audit issue category is incorporated into the reranking input. Experiments are conducted on a Chinese electric power audit text dataset, demonstrating that the proposed retrieval–reranking design provides an effective and practical solution for accurate referenced provision retrieval.

## I. Introduction

**Fig 1:**
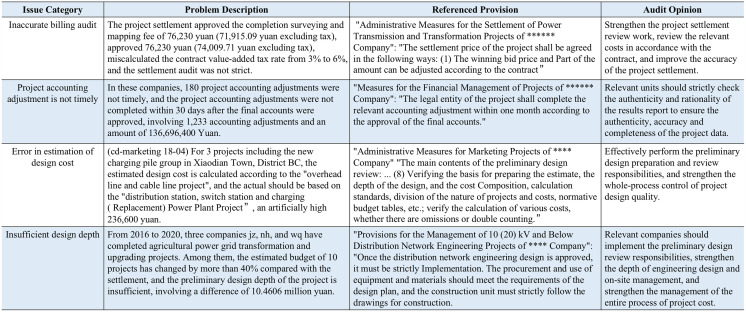
Four anonymized examples of data records in an electric power audit dataset. Each record contains four main fields used in practical audit workflows: audit issue category, problem description, referenced provision, and audit opinion. In typical usage, an audit practitioner writes the problem description (and assigns the issue category), retrieves the most relevant referenced provision from a fixed provision corpus, and then drafts the audit opinion based on the selected provision. All privacy-sensitive information in the shown examples has been desensitized.

Electric power audit plays an important role in electric power systems and directly affects the governance and sustainable development of power enterprises [1,2]. In the process of electric power audit, to record the audit process comprehensively, the auditors usually need to complete four pieces of main data items in the audit form: (i) audit issue category, (ii) problem description, (iii) referenced provision, and (iv) audit opinion, as shown in Fig 1. The audit practitioner should first write a problem description of current audit situation, and then assign a category for it. After that, the practitioner should find a referenced provision from relevant laws and regulations or company rules and regulations. The retrieved referenced provision is finally applied to generate an audit opinion for the enterprise, describing what measures should be taken by the audited enterprise.

Traditionally, the four pieces of data are usually completed manually by audit practitioners. However, these data can actually compete automatically in some cases. For example, audit issue classification and audit opinion drafting have been widely studied in audit-text analytics, and multi-grained pre-trained language models have shown strong performance for electric power audit text classification [2]. Active-learning-based text classification further indicates that annotation-efficient training can be important in specialized domains where labeling is costly [3]. In retrieval-oriented settings, dense retrieval has been shown to improve semantic matching over fixed corpora by learning representation spaces that reduce lexical mismatch [4]. In addition, deep active learning tailored to Chinese power text classification has demonstrated the value of domain-oriented sampling and training strategies [5]. Recently, with the widespread adoption of large language models (LLMs) and instruction/prompt optimization, natural language processing has achieved strong performance across diverse reasoning and generation tasks. Related studies on context-aware prompting optimization [6] and unified text augmentation for dense retrieval [7] suggest that task conditioning and augmentation can improve representation learning; instruction optimization for aspect-based sentiment analysis provides further evidence that guidance signals can improve model behavior in downstream tasks [8]. From an engineering deployment perspective, enterprise decision-support systems also require stability and efficiency under operational constraints, as discussed in AI-driven system identification, control, and optimization research [9]. Prompting multi-task reasoning augmentation further supports the use of auxiliary signals to strengthen task performance under limited supervision [10]. However, in the scenario of electric power audit, the framework of natural language generation (NLG) is not applicable for recommending possible referenced provisions to audit practitioners, as the referenced provisions in laws or regulations are fixed, requiring high precision, which cannot be changed. On the other hand, the generation model has strong generalization ability and uncertainty, so in most of the cases, the generated referenced provisions cannot be matched with existing authoritative clauses.

Prior work relevant to referenced-provision recommendation typically falls into (i) sparse lexical retrieval (e.g., BM25-style matching) [11], (ii) learning-to-rank methods that rely on hand-crafted or shallow features [12,13], and (iii) dense retrieval or pairwise relevance models that improve semantic matching [4]. However, electric power audit provision recommendation has two practical requirements that are not jointly addressed: low-latency retrieval over a fixed provision corpus and high-precision selection of an exact provision text that cannot be altered. This motivates a two-stage design that first performs efficient dense retrieval to recall a small candidate set, and then applies a stronger interaction model for fine-grained reranking. In addition, we explicitly incorporate the audit issue category in the reranking input to reduce ambiguity among semantically similar provisions. These choices distinguish proposed framework from applying a single retriever alone and better align the method with the real audit workflow. Compared with using a single-stage retriever or a full-corpus cross-encoder alone, the novelty of the proposed method is to combine efficient dense recall with category-conditioned reranking to achieve exact provision selection under practical latency constraints.

In computer science, information retrieval (IR) refers to issuing a query and retrieving the most relevant documents from a document set. In early web search, word-frequency and probabilistic matching algorithms such as BM25 were widely used [14]. With the development of deep learning, dense retrieval has become a mainstream approach, where query and document relevance is computed via similarity in a shared embedding space [7]. In modern search systems, IR commonly adopts a two-stage pipeline: (i) retrieval, which recalls a smaller candidate set from a large corpus, and (ii) reranking, which applies stronger interaction modeling to reorder the recalled candidates. Cross-encoder scoring models based on BERT are representative rerankers due to their strong query–document interaction capability, but they are computationally expensive for full-corpus scoring [15]. Recent BERT-based recommenders and matching models further reinforce the effectiveness of cross-encoding for relevance estimation [16], while classical ranking baselines remain important comparators in practical deployments [13], and studies combining BERT with enhanced attention mechanisms highlight continued gains from stronger interaction modeling [17].

Following this IR paradigm, a dense retrieval and reranking framework is proposed for referenced provisions in electric power audit systems. In the retrieval stage, a two-tower (bi-encoder) model encodes the problem description and referenced provision candidates using separate encoders and recalls the top-20 provisions with the highest similarity scores. In the reranking stage, a one-tower (cross-encoder) scoring model jointly encodes the audit issue category, problem description, and each candidate provision to produce fine-grained relevance scores and output the top-1 provision. This two-stage design balances efficiency and accuracy: the retriever supports low-latency recall through offline provision indexing, while the reranker improves precision through deep interaction over a small candidate set.

The proposed design is also motivated by a broader requirement in enterprise AI systems: reliable decision support must remain robust under changing conditions while keeping inference costs bounded. Representation-driven sampling and adaptive policy resetting in multi-agent reinforcement learning provides complementary insights into candidate construction and robustness under complex distributions [18]. Robust disturbance rejection methods also emphasize the importance of maintaining reliable behavior under uncertain inputs [19]. Such considerations are consistent with the motivation for staged pipelines in retrieval, where candidate recall limits computation and reranking focuses modeling capacity on a small set of plausible options [7]. Composite output consensus control for multi-agent systems under heterogeneous disturbances provides another perspective on coordination under non-ideal conditions, which is relevant when audit workflows involve multiple interacting modules (classification, retrieval, and reranking) [20].

A thousand pieces of audit data are collected from a company’s internal audit records, some of whose examples are shown in Fig 1. The “internal audit records” is a document contains an audit issue category, a problem description, a referenced provision selected from a fixed provision corpus, and the resulting audit opinion. The referenced provision in each record corresponds to the provision actually used by practitioners in the original audit workflow; this provision is treated as the ground-truth label for supervised learning and evaluation. To improve reliability, incomplete or inconsistent records were removed and double-check and spot-check verification of provision labels was performed according to a written guideline (e.g., consistency between the described issue and the cited provision). The train/test split is performed at the record level with no overlap, and all methods are evaluated on the same split to ensure fair comparison. Although the provision corpus and audit taxonomy are organization-specific, the retrieval-then-reranking formulation and training procedure are applicable to other electric power audit systems by re-indexing the local provision corpus and fine-tuning the model on local audit records. Before research use, all privacy-sensitive and enterprise-identifying fields were removed or masked (desensitized), while preserving the text necessary for provision retrieval.

The main contributions are summarized as follows:

- A retrieval-then-reranking formulation is established for referenced provision acquisition in electric power audits, explicitly enforcing that outputs are retrieved from an authoritative, fixed provision corpus rather than generated.
- A two-stage dense retrieval framework is developed, combining efficient two-tower recall (top-20) with one-tower reranking for fine-grained semantic matching, and incorporating audit category information as an explicit conditioning signal.
- A desensitized electric power audit dataset is constructed and a comprehensive evaluation against representative sparse retrieval, learning-to-rank, and BERT-based baselines is conducted to validate effectiveness and practicality.

The remainder of this paper is organized as follows: Section II introduces the referenced provision retrieval task and the proposed retrieval models. Section III presents the experimental setup, baselines, evaluation metrics, results, and ablation study. Section IV concludes the paper and outlines future work.

## II. Referenced provision retrieval

### A. Information retrieval for audit

Information retrieval (IR) refers to the process of retrieving relevant items from a corpus in response to a user query, where the items are typically documents or other unstructured content, with the goal of supporting information access and knowledge use. In practical systems, an IR model serves as the interface between users and large repositories (especially text collections) by ranking candidate items according to estimated relevance. IR has been studied for decades, and its importance has increased with the expansion of web-scale search and enterprise knowledge bases. The retrieval target may be heterogeneous, including text, audio, and images; however, audit-oriented provision retrieval is predominantly text-based.

This paper defines the information to be retrieved as unstructured referenced provisions, and the query entered by the user as an audit problem description. In proposed setting, the practitioner query is the free-form problem description written during audit execution, optionally paired with the audit issue category. For example, Issue category: Project settlement; Problem description (query): The project settlement amount exceeds the approved budget and supporting invoices are incomplete. Given this query, the system returns a ranked list of candidate referenced provisions from the fixed provision corpus; we retrieve the top-20 candidates for reranking and output the top-1 provision as the recommended referenced provision. Therefore, the process is similar to calculating the matching score of each candidate referenced provision to the current problem description, and ranking the scores of all provisions from high to low. In order to realize this process, dense retrieval [3,7], which has been proposed in the field of information retrieval in recent years, usually uses the correlation annotation between documents and queries to train a retriever. Then, the generalization ability of the retriever can realize the retrieval and ranking process of any query.

In large-scale retrieval and ranking settings, exhaustive comparison between a query and every document is computationally expensive. A common approach is to pre-encode each document into a vector representation, and at inference time encode the query into the same embedding space. Relevance can then be computed efficiently using vector similarity. This architecture is widely referred to as a two-tower (or dual-encoder) model, where the query encoder and document encoder operate independently and interact only via a similarity function. While two-tower models scale well and enable fast approximate nearest-neighbor search, their independent encoding limits deep token-level interaction between query and document, which can reduce ranking quality.

To improve accuracy, a one-tower (or cross-encoder) model jointly encodes the concatenated query and candidate document and outputs a relevance score. Because the query and document attend to each other within the same encoder, one-tower models typically provide stronger discrimination at the cost of substantially higher computation per query–document pair. Consequently, many practical systems adopt a two-stage retrieval–reranking pipeline: (i) use a two-tower retriever to recall a small candidate set from the full corpus, and (ii) apply a one-tower reranker to refine the ordering within that candidate set.

Following this retrieval–reranking paradigm, this paper first develops a two-tower dense retrieval model for referenced provision retrieval, which recalls the top-20 candidate provisions for each audit query. We then design a one-tower reranking model that incorporates audit issue classification information to support finer-grained relevance estimation and produce the final ranked list of referenced provisions.

### B. Two-tower dense retrieval model for referenced provisions

A two-tower (dual-encoder) retrieval model contains two independent encoders that map the query and the candidate items into a shared embedding space. In text retrieval, these are typically a query encoder and a document encoder; in multimodal retrieval, they may be a text encoder and an image encoder. The main advantage of the two-tower design is computational efficiency at scale. Candidate documents can be encoded offline to form a vector index, and online inference only requires encoding the incoming query once and performing fast vector similarity search against the pre-built index. This “space-for-time” trade-off is a key reason two-tower models are widely deployed in search systems.

**Fig 2:**
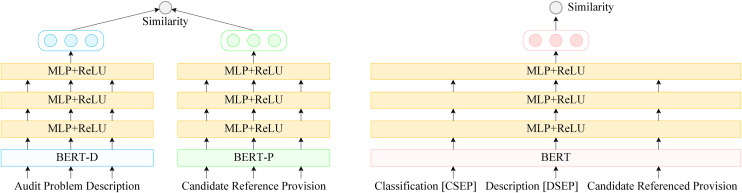
Referenced provision retrieval and reranking framework. The two-tower retriever uses BERT (Bidirectional Encoder Representations from Transformers) encoders with an MLP (multilayer perceptron) projection layer and ReLU (Rectified Linear Unit) activation to retrieve top-20 candidate provisions, followed by a one-tower reranker for fine-grained scoring.

Based on these advantages, this paper first designs a two-tower dense retrieval model to recall potentially relevant referenced provisions from the dataset, as shown in Fig 2(a). The model includes two encoders, BERT-D and BERT-P. BERT-D encodes the current audit problem description, while BERT-P encodes all referenced provisions to be retrieved. The provision encoding can be completed offline in advance.

When a new problem description *d* is input to the model, it is encoded by BERT-D and then passed through a hidden layer HD, which consists of a fully connected layer and a ReLU activation function.


$$f_{d}=ReLU(W_{H}\times BERT-D(d)\;+b_{H})$$ (1)

where ($W_{H}$) denotes the weight matrix of the fully connected layer and ($b_{H}$) denotes its bias vector. In implementation, the one-tower model and the two-tower model employ separate projection heads, so their parameters are not shared. For clarity, these can be written as ($W_{H}^{\left(1\right)}$, $b_{H}^{\left(1\right)}$) for the one-tower model and ($W_{H}^{\left(2\right)}$, $b_{H}^{\left(2\right)}$) for the two-tower model, reflecting the different matching objectives of reranking and retrieval, respectively.

It is also worth noting that multiple hidden layers can be used to enhance the representation capacity of the model. In this paper, we set the projection MLP to three hidden layers. Afterward, similarity is computed between $f_{d}$ and the feature vectors of all candidate referenced provisions in the final scoring stage:


$$sim_{i}=sim\left(f_{d},P_{i}\right)$$ (2)

Finally, the top-20 referenced provisions with the highest similarity scores are selected and passed to the subsequent one-tower reranking model.

### C. One-tower reranking model for referenced provisions

The one-tower model typically fuses the query and document information at the input stage and then feeds the fused representation through stacked hidden layers. This enables the model to learn a deep interaction function between the query and the candidate document and finally output a relevance score. Unlike the two-tower model, the one-tower model needs to run *N* calculations for each new query to obtain relevance scores for the full document set, where *N* is the size of the document set. When *N* is large, this computation becomes prohibitively expensive, which makes full-corpus scoring impractical. However, because the one-tower model captures richer mutual information between the query and the candidate document, it often achieves higher accuracy than the two-tower model in fine-grained ranking.

As shown in Fig 2(b), the one-tower model takes the problem description and a candidate referenced provision as input. To further improve performance, we also incorporate the category corresponding to the current problem into the input (see Fig 1). Specifically, we concatenate the problem classification, problem description, and candidate referenced provision using two special separators, “[CSEP]” and “[DSEP]”. The concatenated sequence is then fed into a BERT encoder for encoding. After that, the representation is passed through the hidden layer HP, and the final relevance score is produced.


$$score=o\left(ReLU\left(W_{H}\times BERT-P\left(C\left[CSEP\right]D\left[DSEP\right]P\right)\;+b_{H}\right)\right)$$ (3)

where *score* denotes the predicted relevance score between the audit problem and the candidate referenced provision. o(.) maps the hidden representation to a scalar output. C denotes the audit problem category label, D denotes the audit problem description, and P denotes a candidate referenced provision. CSEP and DSEP denote the two connector symbols used to construct the one-tower input sequence C[CSEP]D[DSEP]P. This concatenated sequence is jointly encoded by the one-tower encoder BERT−P(.), producing a contextual representation that is then passed through the fully connected layer with ReLU activation and finally through o(.) to obtain *score*. For clarity, the two-tower retriever encodes D and P separately using BERT−D and BERT−P for efficient candidate retrieval, whereas the one-tower model performs joint encoding over C[CSEP]D[DSEP]P for fine-grained reranking; the parameters of the reranker are not shared with the two-tower encoders.

In this setting, for each new problem description, the one-tower model is executed only on the recalled candidate set. Therefore, the model is computed 20 times and then reranks the candidate referenced provisions based on the relevance scores from the output layer.

## III. Experiments

### A. Details

The Merlin Models library is used to implement both the two-tower retrieval model and the one-tower scoring model. Merlin Models is built on TensorFlow and provides modular components for constructing text matching architectures, including the two-tower retrieval and one-tower scoring pipelines adopted in this work.

For the two-tower retrieval model, categorical cross-entropy is used as the loss function and Adam is applied for optimization, with the learning rate kept at the library default setting. The batch size is set to 8. For the one-tower scoring model, the same optimizer configuration and batch size are used. During training of the scoring model, negative samples are critical. Negative samples are generated using the UniformNegativeSampling class provided in Merlin Models. The loss function for the one-tower scoring model is binary cross-entropy. The collected dataset is split into training and test sets with a 4:1 ratio. Training is performed on the training set, and all evaluation metrics are computed on the test set.

The experiments were conducted on a GPU cloud server with an Intel(R) Xeon(R) Silver 4114 CPU @ 2.20 GHz, four NVIDIA Titan V GPUs (12 GB VRAM each), 256 GB RAM, and a 2 TB disk. We benchmarked online inference over *N* = 200 test queries after 20 warm-up queries, using batch size 1 for two-tower retrieval and batch size 20 for one-tower reranking. The average end-to-end latency is 23 ms per query, consisting of 4 ms for retrieving the top-20 candidates and 19 ms for reranking the 20 candidates, corresponding to approximately 43 queries per second. Offline provision indexing is performed once before online use and takes about 10 s in proposed setting.

### B. Baseline retrievers

To evaluate the effectiveness of the proposed retrieval framework, several widely used baselines in information retrieval and learning-to-rank are implemented for comparison, including the frequency-based retriever BM25, neural learning-to-rank methods RankNet and LambdaMART, and a dense retriever based on BERT.

- BM25 [11,14]: BM25 is a probabilistic term-matching method that ranks documents by their lexical relevance to a query. Given a query q and a document collection D, BM25 assigns a score to each document d ∈ D by aggregating term-level contributions. These contributions reflect the term frequency within the document, inverse document frequency across the corpus, and document-length normalization, which together improve robustness across varying document lengths.
- RankNet [12]: RankNet is a pairwise learning-to-rank model that learns ordering preferences between document pairs (di, dj) by modeling the probability that di should be ranked ahead of dj. In this work, RankNet is instantiated as a fully connected neural network, and the model is optimized using a probability-based cross-entropy objective over pairwise preference labels.
- LambdaMART [13]: LambdaMART combines boosted decision trees with the LambdaRank idea to directly optimize ranking quality. Instead of explicitly constructing and differentiating a potentially ill-formed cost function, LambdaMART defines “lambda” gradients that are shaped by both pairwise ranking errors (as in RankNet) and listwise ranking metrics (typically NDCG). This design enables effective training toward ranking performance while retaining the scalability of gradient-boosted trees.
- BERT [15]: The BERT baseline fine-tunes a pre-trained BERT model with an additional feed-forward network to predict a relevance score for each query–provision pair. During inference, the referenced provision with the highest predicted score among the candidate list is selected.

Table 1 summarizes the key similarities and differences between the proposed retrieval variants (Two-tower + One-tower, w/o One-tower, and w/o Classification) and the baseline methods, in terms of interaction pattern, offline indexing, and online cost.

**Table 1:** Summary of Baseline retriever properties.

Method	Family	Interaction pattern	Offline indexing	Online cost per query	What it mainly shares/differs
BM25	Sparse lexical retrieval	Token overlap scoring	Yes	Low	Fast lexical baseline over the same provision corpus
RankNet	Learning-to-rank	Pairwise scoring using engineered/text features	No (typical)	Medium	Supervised LTR baseline; ranking function differs from dense semantic retrieval
LambdaMART	Learning-to-rank	Tree-based ranking using engineered/text features	No (typical)	Medium	Strong classical LTR baseline; differs in model family and feature dependence
BERT (scratch)	Dense one-tower scoring	Cross-encoding query–provision pairs	No	High	Strong interaction model but must score many pairs; no retrieval index
BERT (pretrain)	Dense one-tower scoring	Cross-encoding query–provision pairs	No	High	Same as above but pretrained initialization; typically higher accuracy
Proposed (Two-tower + One-tower)	Two-stage dense retrieval + reranking	Bi-encoder retrieve top-K + cross-encoder rerank	Yes	Medium	Efficient retrieval via indexed provision embeddings, then rerank only top-20
Proposed (w/o One-tower)	Dense retrieval only	Bi-encoder (two-tower)	Yes	Low	Fastest dense variant; validates reranking contribution
Proposed (w/o Classification)	Two-stage dense retrieval + reranking	Same as proposed, without category input	Yes	Medium	Validates contribution of audit category information

### C. Evaluation metrics

- Precision@k (*P@k*): This metric quantifies how many items in the top-K results were relevant. Mathematically, this is given by:

$$P@k=\frac{TruePositives@k}{TruePositives@k+FalsePositives@k}$$ (4)

- Mean Reciprocal Rank (MRR): This metric is useful when the proposed system is expected to return the most relevant item and rank it as highly as possible. Mathematically, this is given by:

$$MRR=\frac{\text{1}}{Q}\sum_{i=1}^{\left|Q\right|}\frac{\text{1}}{rank_{i}}$$ (5)

- Mean Average Precision (MAP): If we want to evaluate average precision across multiple queries, we can use the MAP. It is simply the mean of the average precision for all queries. Mathematically, this is given by:

$$MRP=\frac{\text{1}}{\left|Q\right|}\sum_{q=1}^{\left|Q\right|}AP\left(q\right)$$ (6)

where MRP represents the average of the average precision of the relevance scores obtained for all audit issue descriptions, and this average value is used to represent the average precision of the relevance scores. Q represents the total number of the audit problem description input. *AP*(*q*) is the average precision for query q which can be calculated as:


$$AP=\frac{\sum_{i=1}^{k}P@i\times rel\left(i\right)}{number\;of\;relevant\;items}$$ (7)

where k represents a parameter that indicates the metric to be used for measuring the top k results. i represents the document at the i -th position. rel(i) represents the document relevance at the i -th position, it is an indicator function which is 1 when the item at rank k is relevant. P@i represents accuracy rate@i, and @i represents the total numerical value of all the stated correlation scores.

- Normalized Discounted Cumulative Gain (NDCG@k): To allow a comparison of discounted cumulative gain (DCG) across queries, we can use NDCG that normalizes the DCG values using the ideal order of the relevant items:

$$NDCG@k=\frac{DCG@k}{IDCG@k}$$ (8)

where NDCG@k represents the normalized discounted cumulative gain. DCG@k represents discounted cumulative gain. IDCG@k represents denotes the ideal DCG@k.

we can calculate the DCG simply by taking the sum of the relevance score normalized by the penalty:


$$DCG@k=\sum_{i=1}^{k}\frac{rel\left(i\right)\;}{log_{\text{2}}i+1}$$ (9)

### D. Experimental results and analysis

The two-tower retrieval model and the one-tower scoring model are trained on the training set, with 10% of the training data held out as a validation set. During training, the validation *P@1* is monitored for early stopping. After one epoch, the validation *P@1* no longer improves; training is then stopped and all evaluation metrics are computed on the test set to obtain the final results.

The results support the following observations:

- First, compared with traditional retrieval and learning-to-rank baselines (BM25, RankNet, and LambdaMART), the proposed approach achieves better performance across all evaluation metrics. This indicates that dense retrieval combined with reranking is effective for referenced provision retrieval. In particular, the learning-to-rank baselines (RankNet and LambdaMART) substantially outperform the frequency-based BM25 baseline, and the BERT-based model further improves over RankNet and LambdaMART.
- Second, based on P@1, the top-1 retrieved provision matches the ground truth in approximately 43% of cases. This suggests that the model can directly provide the correct referenced provision in nearly half of the instances. In practice, an audit practitioner can directly adopt the top-1 provision when high precision is required.
- Third, the NDCG@5, NDCG@10, and NDCG@20 results indicate that even when the top-1 provision is not the only correct match, the correct referenced provision is often covered within the top-k returned candidates (k = 5, 10, 20). In this setting, the model provides a short list of highly plausible provisions, which can significantly reduce the manual search scope for audit practitioners.

### E. Ablation study

An ablation study is conducted to quantify the contribution of each module in the proposed method.

First, to assess the benefit of the retrieval-then-reranking framework, the one-tower scoring model is removed, denoted as “w/o. One-tower” in Table 2. In this variant, evaluation is performed directly using the ranking produced by the two-tower retriever. The results show a significant drop across all metrics, indicating that reranking provides substantial gains and that the two-stage retrieval–reranking framework is more effective than using retrieval alone.

**Table 2:** Performance comparison on the chinese electric power audit text dataset.

Method	*P@1*	MAP	MRR	*NDCG@1*	*NDCG@5*	*NDCG@10*	*NDCG@20*
BM25	0.1227	0.1476	0.1733	0.1062	0.1282	0.1254	0.1203
RankNet	0.2135	0.2590	0.2981	0.1911	0.2231	0.2246	0.2190
LambdaMART	0.2644	0.3061	0.3266	0.2108	0.2252	0.2247	0.2213
BERT	0.3736	0.3847	0.3939	0.2784	0.2831	0.2790	0.2794
Proposed (Two-tower + One-tower)	**0.4309**	**0.4486**	**0.4607**	**0.3200**	**0.3238**	**0.3231**	**0.3211**
w/o. One-tower	0.4055	0.4174	0.4332	0.2981	0.3030	0.3017	0.3009
w/o. Classification	0.4137	0.4280	0.4395	0.3064	0.3099	0.3071	0.3036

Second, the audit problem classification information is removed from the one-tower scoring model, denoted as “w/o. Classification” in Table 2. In this case, all metrics also decrease, confirming that audit category information contributes to reranking performance. However, the degradation is smaller than that observed when removing the one-tower model, indicating that the reranking stage is the dominant contributor, while category information provides an additional, complementary improvement.

We further ablate key design choices within each stage. First, we vary the number of hidden layers in the projection MLP of the two-tower encoders (e.g., 1/2/3/4 layers) while keeping other settings fixed, to quantify how representation capacity affects retrieval quality. Second, we compare different pretrained BERT checkpoints for the two-tower and one-tower encoders (e.g., base vs domain-adapted variants if available) to measure sensitivity to backbone selection. We report the same ranking metrics and additionally summarize the associated inference-time change, providing a clearer optimization direction for model structure under practical latency constraints.

## IV. Conclusion

Referenced provision retrieval is a critical yet challenging step in electric power audits, where an audit opinion must be supported by the most relevant provision from a large corpus under strict correctness and traceability requirements. This paper addressed the problem by proposing a dense retrieval–reranking framework that combines a two-tower retriever for efficient top-20 recall with a one-tower reranker for fine-grained semantic matching, further enhanced by incorporating audit category information to improve context-aware discrimination. Experiments on a Chinese electric power audit dataset demonstrate strong effectiveness, achieving *P@1* = 0.4309, MAP = 0.4486, MRR = 0.4607, and stable ranking quality (e.g., *NDCG@5* = 0.3238, *NDCG@20* = 0.3211); ablation results confirm the value of reranking (*P@1*: 0.4309 → 0.4055 without one-tower) and the additional benefit of category information (*P@1*: 0.4309 → 0.4137 without classification). Future work can focus on stronger hard-negative sampling and domain-adaptive training to improve robustness on semantically similar provisions, multi-task joint learning of classification and reranking, and broader validation on larger cross-domain audit corpora to support deployment in real audit systems.
